# Comment on: “Objectification of Skin Glow: In Vivo Evaluation of 300 Women in Relation to Age”

**DOI:** 10.1111/jocd.70539

**Published:** 2025-11-09

**Authors:** Moeeza Fatima, Husnain Ahmad, Fatima Qasim, Isha Munir

**Affiliations:** ^1^ Shaikh Khalifa Bin Zayed Al‐Nahyan Medical College Shaikh Zayed Postgraduate Medical Institute (SZPGMI) Lahore Pakistan; ^2^ Ch Pervaiz Elahi Institute of Cardiology Multan Pakistan; ^3^ Shaikh Khalifa Bin Zayed Al‐Nahyan Medical College Shaikh Zayed Postgraduate Medical Institute (SZPGMI) Lahore Pakistan; ^4^ Shaikh Khalifa Bin Zayed Al‐Nahyan Medical College Shaikh Zayed Postgraduate Medical Institute (SZPGMI) Lahore Pakistan

Dear Editor,

We read with great interest the recent article by Roessle and Kerscher, which represents a noteworthy contribution towards understanding and objectifying the concept of skin glow [1]. Their in vivo evaluation of 300 women in relation to age offers valuable insights into this complex trait. While the study provides proper preliminary reference values, several limitations were not sufficiently addressed and warrant further consideration to improve the applicability and generalizability of the findings.

First, while the study restricts participants to Fitzpatrick skin types I–IV, the exclusion of individuals with **darker phototypes** significantly limits the ability to generalize the findings. Ethnic differences in skin characteristics, such as melanin distribution, vascular reactivity, and light scattering properties, can affect how skin glow is perceived and measured. Studies have shown that darker skin types exhibit distinct patterns of pigmentation and erythema [2, 3]. Given that this study is based on a narrow range of phototypes, the conclusions may not apply to broader, more diverse populations. Since this is a short study with an exploratory design, it is crucial to acknowledge the ethnic limitations and suggest that future studies include a wider range of skin types to make the findings more inclusive and applicable.

Second, the study's exclusive focus on **female participants** limits the applicability of the results. While sex differences in skin physiology are well documented (particularly in terms of sebum secretion and vascular activity), the exclusion of males from this study leaves a gap in understanding how these findings may translate across genders [4]. This limitation should be addressed, as it reduces the external validity of the study. Short studies, such as the one presented, typically have more targeted samples. Still, it is critical to highlight the importance of including both males and females in future research to ensure balanced representation.

Third, the anatomical sites measured may not optimally reflect clinically relevant **glow‐related changes**. Lateral cheek measurements, for example, may underestimate centrofacial aging patterns, where pigmentation and telangiectasia are most pronounced [5]. Similarly, neck and décolleté readings are influenced by clothing and cultural behaviors, limiting universal applicability. Lastly, the study omitted molecular and structural markers that strongly influence glow. Studies have shown that advanced glycation end‐products (AGEs), collagen integrity, and oxidative stress biomarkers are known to alter skin radiance independent of erythema or melanin indices [6, 7]. Their exclusion narrows the biological interpretation of the findings.

Looking forward, several advancements could address these gaps. High‐resolution imaging modalities, such as **hyperspectral imaging** and **AI‐driven skin analysis**, can now quantify complexion heterogeneity and radiance more precisely [8]. In vivo Raman spectroscopy allows non‐invasive detection of AGEs, offering mechanistic insights. On the therapeutic front, next‐generation hybrid fillers (e.g., CPM‐HA20G) and biostimulatory injectables have shown sustained improvements across multiple skin quality domains, including glow [9]. Integrating these approaches with lifestyle‐adjusted, longitudinal data would provide a more comprehensive and clinically meaningful framework.

While Roessle and Kerscher provide valuable preliminary benchmarks for skin glow, the study's limited phototype range, female‐only cohort, single‐visit design, assumptions regarding missing data, site‐selection issues, lack of reproducibility testing, and omission of mechanistic biomarkers constrain its interpretability and generalisability. Future studies should adopt broader, longitudinal, and multimodal approaches to establish robust, inclusive, and clinically relevant standards for skin glow assessment.

## Author Contributions

Moeeza Fatima and Husnain Ahmad conceived the idea, reviewed the original article, performed the literature search, and drafted the manuscript. Fatima Qasim and Isha Munir reviewed and approved the final version of the letter.

## Ethics Statement

The authors have nothing to report.

## Consent

The authors have nothing to report.

## Conflicts of Interest

The authors declare no conflicts of interest.

## Linked Articles

This article is linked to Roessle and Kerscher paper. To view this article, visit https://doi.org/10.1111/jocd.70373.

## Data Availability

The authors have nothing to report.
